# Exploring MRI based radiomics analysis of intratumoral spatial heterogeneity in locally advanced nasopharyngeal carcinoma treated with intensity modulated radiotherapy

**DOI:** 10.1371/journal.pone.0240043

**Published:** 2020-10-05

**Authors:** Farhan Akram, Ping En Koh, Fuqiang Wang, Siqin Zhou, Sze Huey Tan, Mahsa Paknezhad, Sojeong Park, Tiffany Hennedige, Choon Hua Thng, Hwee Kuan Lee, Kiattisa Sommat

**Affiliations:** 1 A*STAR (Agency for Science, Technology and Research), Bioinformatics Institute, Singapore; 2 Divisions of Radiation Oncology, National Cancer Centre Singapore, Singapore; 3 Divisions of Clinical Trials and Epidemiological Sciences, National Cancer Centre Singapore, Singapore; 4 Divisions of Oncologic Imaging and Epidemiological Sciences, National Cancer Centre Singapore, Singapore; Northwestern University Feinberg School of Medicine, UNITED STATES

## Abstract

**Background:**

We hypothesized that spatial heterogeneity exists between recurrent and non-recurrent regions within a tumor. The aim of this study was to determine if there is a difference between radiomics features derived from recurrent versus non recurrent regions within the tumor based on pre-treatment MRI.

**Methods:**

A total of 14 T4NxM0 NPC patients with histologically proven “in field” recurrence in the post nasal space following curative intent IMRT were included in this study. Pretreatment MRI were co-registered with MRI at the time of recurrence for the delineation of gross tumor volume at diagnosis(GTV) and at recurrence(GTVr). A total of 7 histogram features and 40 texture features were computed from the recurrent(GTVr) and non-recurrent region(GTV-GTVr). Paired t-tests and Wilcoxon signed-rank tests were carried out on the 47 quantified radiomics features.

**Results:**

A total of 7 features were significantly different between recurrent and non-recurrent regions. Other than the variance from intensity-based histogram, the remaining six significant features were either from the gray-level size zone matrix (GLSZM) or the neighbourhood gray-tone difference matrix (NGTDM).

**Conclusions:**

The radiomic features extracted from pre-treatment MRI can potentially reflect the difference between recurrent and non-recurrent regions within a tumor and has a potential role in pre-treatment identification of intra-tumoral radio-resistance for selective dose escalation.

## Background

[1–4] Approximately one third of patients with locally advanced nasopharyngeal cancer (NPC) still developed local recurrence after curative dose of intensity modulated radiation therapy (IMRT) and chemotherapy. The management of local recurrence remains a challenging issue for radiation oncologists as salvage treatment options for locally recurrent NPC offer limited local control and survival benefits with high toxicities. [5,6] The majority of these local recurrences were observed to be ‘in field’, occurring within the high radiation dose region, suggesting the presence of sub-populations of cancer cells within the tumor which are radio-resistant. [7–10] The development of subpopulations of cancer cells with divergent biological behavior within a primary tumor is also known as intra-tumoral heterogeneity. [11–14] It is now well established from a variety of studies that tumors with significant intra-tumoral heterogeneity are associated with more aggressive behavior, poorer prognosis and resistance to radiotherapy.

[15–17] The past decade has seen rapid developments in medical imaging that provide better spatial resolution and allow for assessment of tumor heterogeneity. [18–20] Emerging data showed that these modalities hold the potential to identify regions within tumors which are radio-resistant. [21] Escalating the dose to the entire gross tumor volume (GTV) may not always be possible as the dose to the adjacent critical normal tissues will inevitably also increase, leading to complications. It was therefore suggested that dose escalation should be targeted to areas of increased radio-resistance in the tumor. [22–26] Radiomics is an emerging field that extracts a large amount of quantitative features from imaging scans in order to characterize intra-tumoural heterogeneity and to reveal important prognostic information about the cancer. These radiomics features have the potential to unravel disease characteristics that could be missed by the naked eye. The advances in functional and spatial imaging, coupled with radiomics, allow for “dose painting” that consists of selective dose escalation to regions within the tumor with relative radio-resistance. [27–29] As such, there is a growing interest in “dose painting” which may be an effective way to reduce “in field” recurrence with acceptable side effects.

[30, 31] Magnetic resonance imaging (MRI) is the imaging of choice in the diagnosis and local staging in NPC due to its superior soft tissue contrast and allows for accurate delineation of target volumes for purposes of radiotherapy. [32–37] Whilst some research has been carried out on the application of radiomics in nasopharyngeal cancer, an approach that utilizes MRI radiomics as a predictive signature for intra-tumoral radio-resistance has not yet been developed. Comprehensive image analysis using radiomics that can identify radio-resistant tumor sub-volumes from pre-treatment MRI scans could guide individualized radiation therapy by suggesting target volumes in which a higher dose of radiation is needed for better tumor control. We hypothesized that spatial heterogeneity exists between recurrent and non-recurrent regions within a tumor. Therefore, the aim of this study was to determine if there is a difference between radiomics features derived from recurrent versus non recurrent regions within the tumor based on pre-treatment MRI.

## Methods and materials

This is a retrospective review of patients with AJCC 7th Edition T4, Nx and non metastatic nasopharyngeal carcinoma treated with curative intent intensity modulated radiotherapy in our Department of Radiation Oncology in National Cancer Centre Singapore between January 2010 and December 2012. Out of the 87 patients treated within this period, a total of 14 patients who developed histologically proven recurrence in the nasopharynx were included in this study. This study was approved by Singhealth institutional review board and the requirement for informed consent was waived.

**Follow up** After treatment, patients were followed up every 2 months in the first year, every 4 months in the second year and every 6 months from the third to fifth year, then yearly afterwards. Locoregional imaging with contrast-enhanced CT, or MRI or PET-CT scans were requested routinely on a yearly basis and/or earlier if clinically indicated.

### MRI imaging protocol

Pre-treatment and recurrent MRI scan of the skull base and neck were performed with a 1.5 Tesla scanner (GE Signa Echospeed; GE Medical Systems, Milwaukee, USA). Gadopentetate dimeglumine (Magnevist; Schering Diagnostics AG, Berlin, Germany) at 0.1 mmol/kg of body weight was administered and images of the patients in their customized radiotherapy immobilization shell were acquired. The reconstructed voxel size was 0.7 x 0.7 x 5 mm^3^. Out of the various MRI sequences, we have selected the contrast-enhanced fat-suppressed T1-weighted spin echo sequences in axial planes (CE FS axial T1) for analysis. [38,39] CE FS axial T1 has been consistently reported as the most informative individual sequence in delineating the local extent of NPC.

### Image acquisition, segmentation and radiomic feature extraction

**Fig 1:**
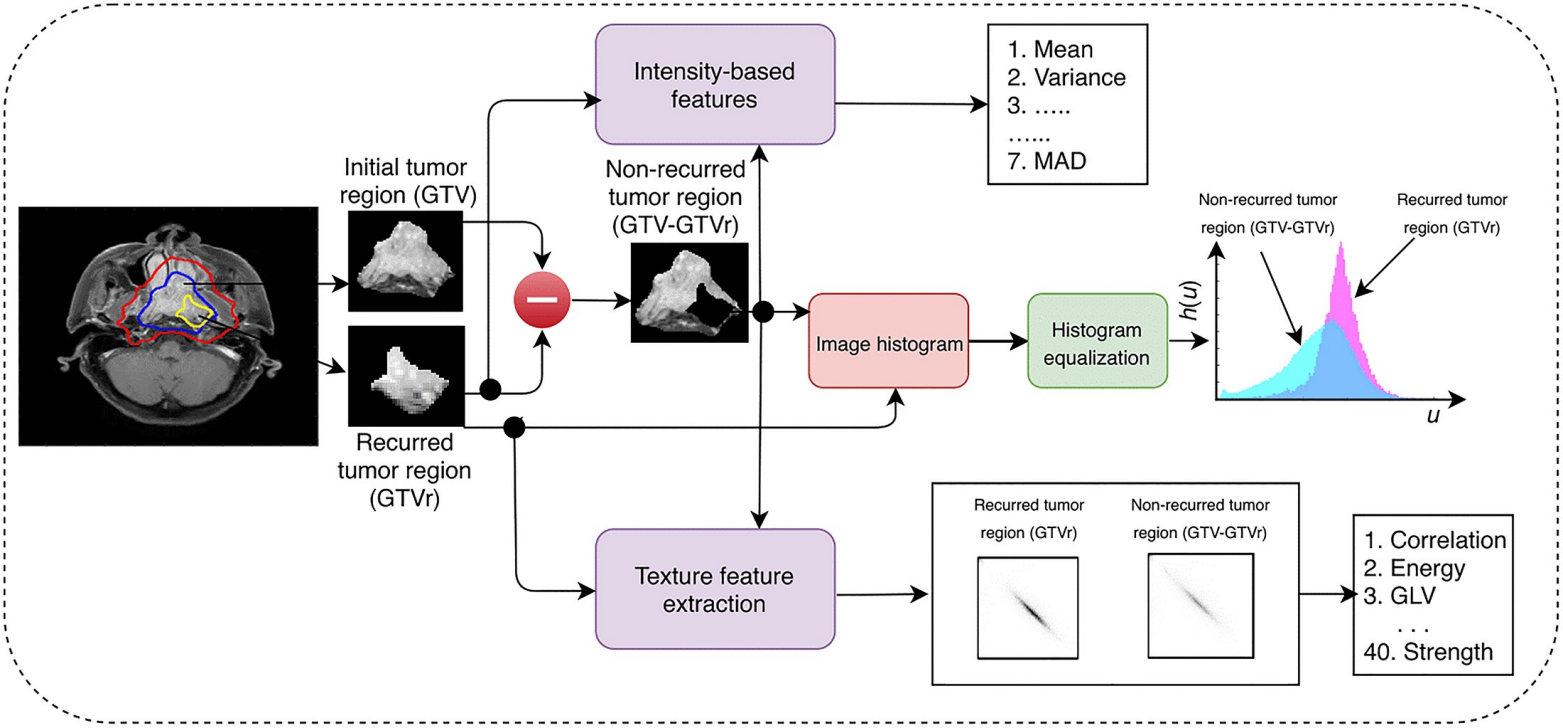
Radiomics workflow.

Fig 1 showed the radiomics workflow. All patients had MRI scans at pre-treatment and at the time of local recurrence. The fusion of pre-treatment MRI with CT simulation images was routinely performed using rigid registration for all patients for accurate delineation of gross tumor volumes (GTV) and critical structures. All GTVs were contoured at the time of diagnosis. These fused pre-treatment MRI and CT images together with the GTV contours and 95% isodose lines were retrieved from Eclipse Treatment Planning System. MRI images at the time of local recurrence were then fused with the CT simulation images. The recurrent gross tumor volume (GTVr) was identified and contoured in retrospect on MRI images at the time of local recurrence by a single expert radiation oncologist. The structures GTVr, GTV and 95% isodose lines were copied from the CT simulation images onto the pretreatment CE FS axial T1 MRI (S1 Appendix). [40] We adopted the definition of “in field” recurrence from previously published reports as 95% of recurrent gross tumor volume (GTVr) was within the 95% isodose. Each pre-treatment CE FS axial T1 MRI contained two regions of interest (ROI), which were the recurrent region (GTVr) and the non-recurrent region (GTVnr), which was obtained by subtracting the overlapping GTVr from the GTV (GTV-GTVr) (S2 Appendix). X and Y dimensions of the input MRI images are 512x512 while the number of slices, i.e., Z dimension is varying for all patients (ranges from 44 to 100). Radiomics features were then extracted from these two ROI:

- Intensity Based Histogram
  Intensity-based histograms were computed for both recurrent and non-recurrent regions. The histograms of both regions were then normalized on the same intensity scale for a fair comparison between their distributions. On the basis of the first-order statistic, 7 histogram features were computed for both recurrent and non-recurrent tumor volumes: mean, variance, skewness, kurtosis, mean absolute deviation (MAD), hyperskewness and hyperflatness.
- Texture Analysis
  An open source radiomics library (https://github.com/mvallieres/radiomics/tree/master/TextureToolbox) was used to compute different texture features for both recurrent and non-recurrent regions, such as gray-level co-occurrence matrix (GLCM), gray-level run length matrix (GLRLM), gray-level size zone matrix (GLSZM) and neighborhood gray-tone difference matrix (NGTDM) texture matrices. [41] The recurrent (GTVr) and non-recurrent tumor regions (GTV-GTVr) were prepared by using Lloyd-Max quantization algorithm with the quantization level of 256 as the intensity gray-level in the pretreatment MRI is 256. A total of 40 texture features were computed from 4 texture metrics: GLCM, GLRLM, GLSZM, and NGTDM, where the texture metrics contained 9, 13, 13 and 5 features, respectively (Table 1).

**Table 1:** 47 texture features from intensity histogram and texture metrics: GLCM, GLRLM, GLSZM and NGTDM.

Texture metrics	Features
Intensity Histogram	Mean, variance, skewness, kurtosis, mean absolute deviation (MAD), hyperskewness, hyperflatness
GLCM	Energy, contrast, entropy, homogeneity, correlation, sum average, variation, dissimilarity, auto correlation
GLRLM	Short run emphasis (SRE), long run emphasis (LRE), gray level non-uniformity (GLN), run length non-uniformity (RLN), run percentage (RP), low gray-level run emphasis (LGRE), high gray-level run emphasis (HGRE), short run low gray-level emphasis (SRLGE), short run high gray-level emphasis (SRHGE), long run low gray-level emphasis (LRLGE), long run high gray-level emphasis (LRHGE), gray level variance (GLV), run length variance (RLV)
GLSZM	Small zone emphasis (SZE), large zone emphasis (LZE), gray-level non-uniformity (GLN), Zone size non-uniformity (ZSN), zone percentage (ZP), low gray-level zone emphasis (LGZE), high gray-level zone emphasis(HGZE), small zone low gray-level emphasis (SZLGE), small zone low gray-level emphasis (SZHGE), large zone low gray-level emphasis (LZLGE), large zone high gray-level emphasis (LZHGE), gray level variance (GLV), zone size variance (ZSV)
NGTDM	Coarseness, contrast, busyness, complexity, strength

### Statistical analysis

Shapiro-Wilk normality tests were carried out on the differences between GTVr and GTV-GTVr pairs for the 47 features, and p-values < 0.05 were considered significantly different. Paired t-tests were performed on the features and Wilcoxon signed-rank tests were carried out on the features that violated the normality assumption. Applying Bonferroni correction, p-values less than 0.05/47 = 0.001 were considered as statistically significant. Pearson Correlation Coefficient (PCC) values with p-values were computed for the paired differences of the recurrent and non-recurrent regions for the significant features. Principal component analysis (PCA) was performed on significant features to reduce dimensionality. All statistical analyses were performed using R software (version 3.6.0).

## Results

Out of the 87 T4NxM0 NPC patients treated in National Cancer Centre Singapore between January 2010 and December 2012, 14 patients had developed histologically proven recurrence and had both the pre-treatment and recurrent MRI scans available. The 14 recurrent MRI scans were fused with the corresponding pre-treatment MRI scan to segregate the initial tumor into two regions of interest, the recurrent and non-recurrent regions. A total of 7 histogram features and 40 texture features were computed for the regions of interest. Paired t-tests and Wilcoxon signed-rank tests were carried out on the 47 quantified radiomics features, and 7 features were statistically significant at p-values < 0.001 as shown in Table 2. Other than variance from the intensity-based histogram, the remaining six significant features were either from the gray-level size zone matrix (GLSZM) or the neighbourhood gray-tone difference matrix (NGTDM).

**Table 2:** Features show statistical difference between GTVr and GTV-GTVr regions.

Features(n = 47)	Mean of the differences (TR vs TMTR)	SD of the differences (TR vs TMTR)	P-value
GLSZM_GLV	4.06E-02	3.00E-02	0.0002
GLSZM_ZSV	1.98E-06	1.49E-06	0.0003
GLSZM_LZE	-3.63E-01	5.17E-01	0.0009^
Intensity_Variance	-5.38E+02	4.38E+02	0.0005
NGTDM_Busyness	-5.31E-02	2.94E-02	<0.0001
NGTDM_Coarseness	3.39E-03	2.59E-03	0.0003
NGTDM_Strength	5.15E+01	4.10E+01	0.0004

p-value calculated using paired t-test unless otherwise stated.

^p-value calculated using Wilcoxon signed-rank test.

**Fig 2:**
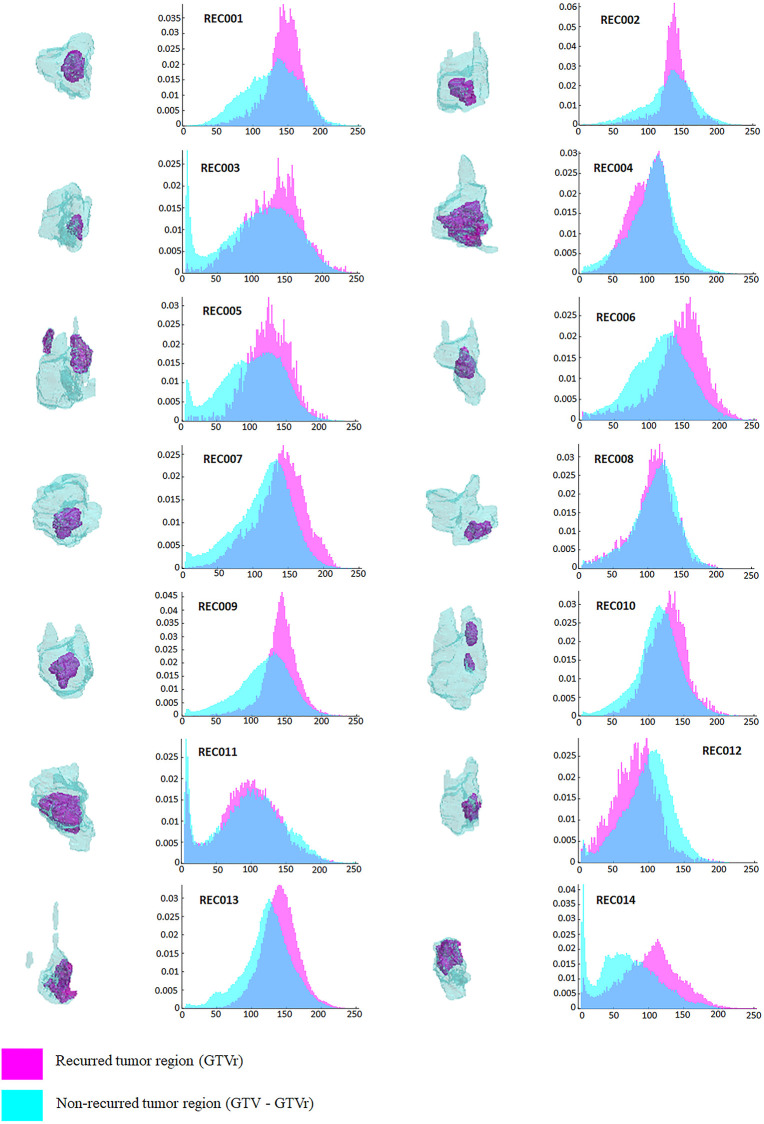
Intensity-based histogram comparison between recurrent (GTV) and non-recurrent regions (GTV-GTVr) in all patients (n = 14, REC001-REC014).

**Fig 3:**
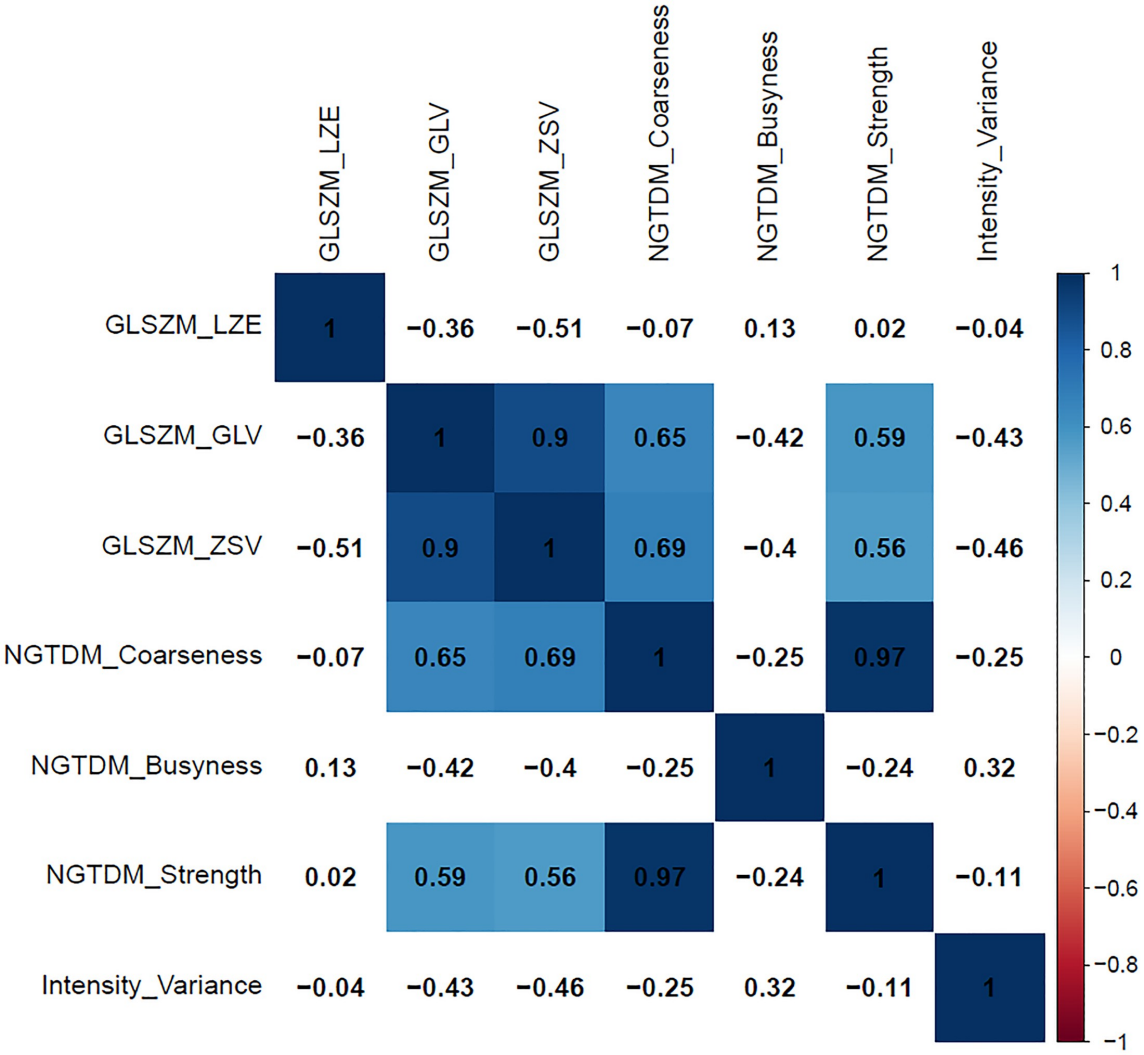
Pearson correlation coefficient matrix on the paired differences of the seven significant features.

Fig 2 showed the intensity histograms generated from the recurrent and non-recurrent regions within each tumor volume for each patient. Visually, 11 (all patients except REC004, REC008 and REC011) out of 14 histograms had bigger peaks on the high intensity bins for recurrent tumor regions compared to the non-recurrent tumor regions. Fig 3 displayed the Pearson correlation coefficients for the paired differences between the recurrent and non-recurrent regions. Correlation coefficients with p-values < 0.05 were considered significant and colored according to the color bar, while insignificant correlation coefficients were not coloured. As shown in Fig 3, none of the negative Pearson correlation coefficients were significant, and positive correlation with p-values < 0.05 were observed among GLSZM_GLM, GLSZM_ZSV, NGTDM_Coarseness and NGTDM_Strength.

**Fig 4:**
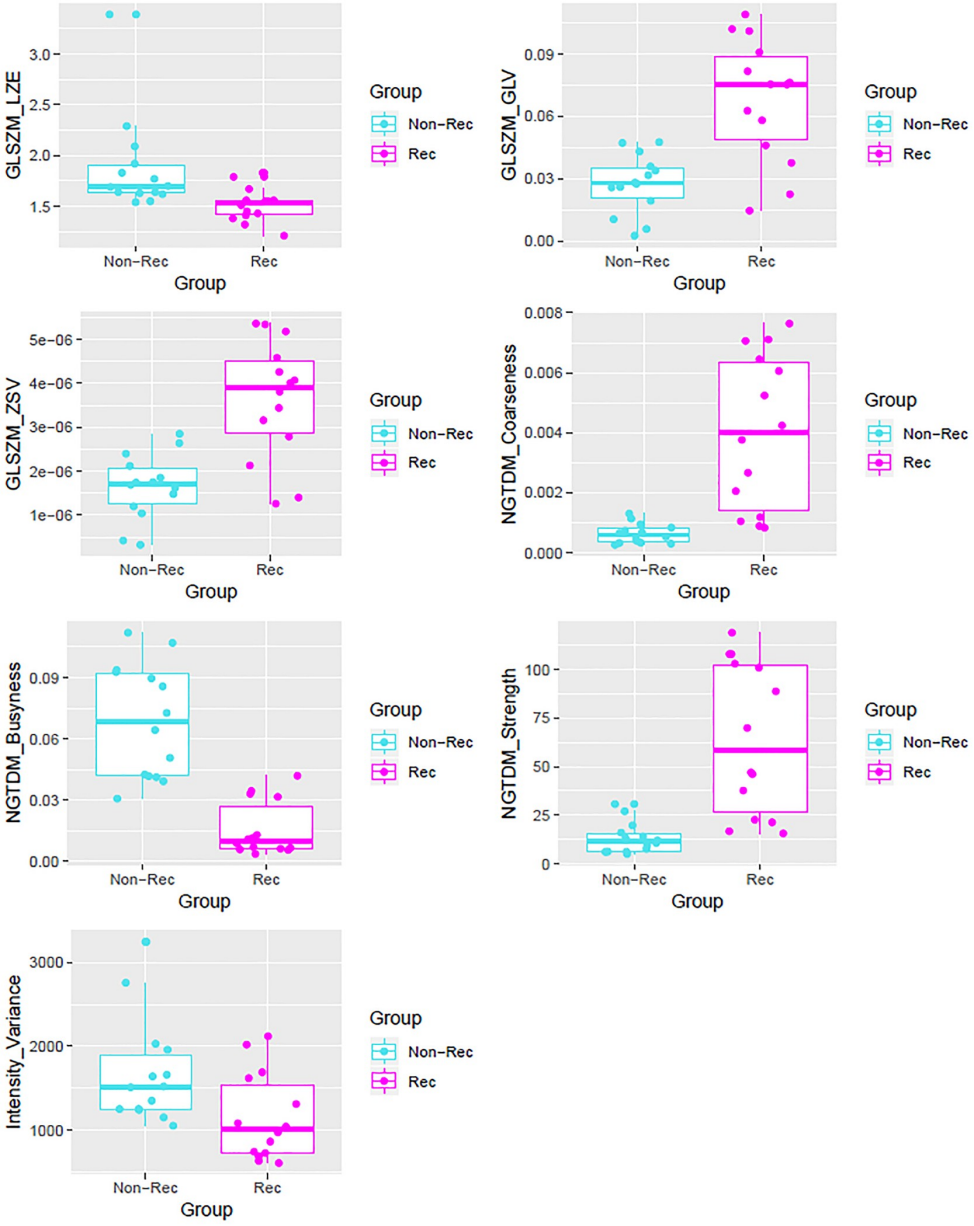
Box plots of the significant features, differentiating the recurrent and the non-recurrent regions.

Fig 4 displayed the distribution of the selected features. As shown by the box plots in Fig 4, busyness, coarseness and strength from NGTDM were performing well in differentiating the recurrent and non-recurrent regions. And given NGTDM busyness was not significantly correlated with any other feature in Fig 3, it could potentially serve as an independent parameter in predicting the recurrent and non-recurrent region.

**Fig 5:**
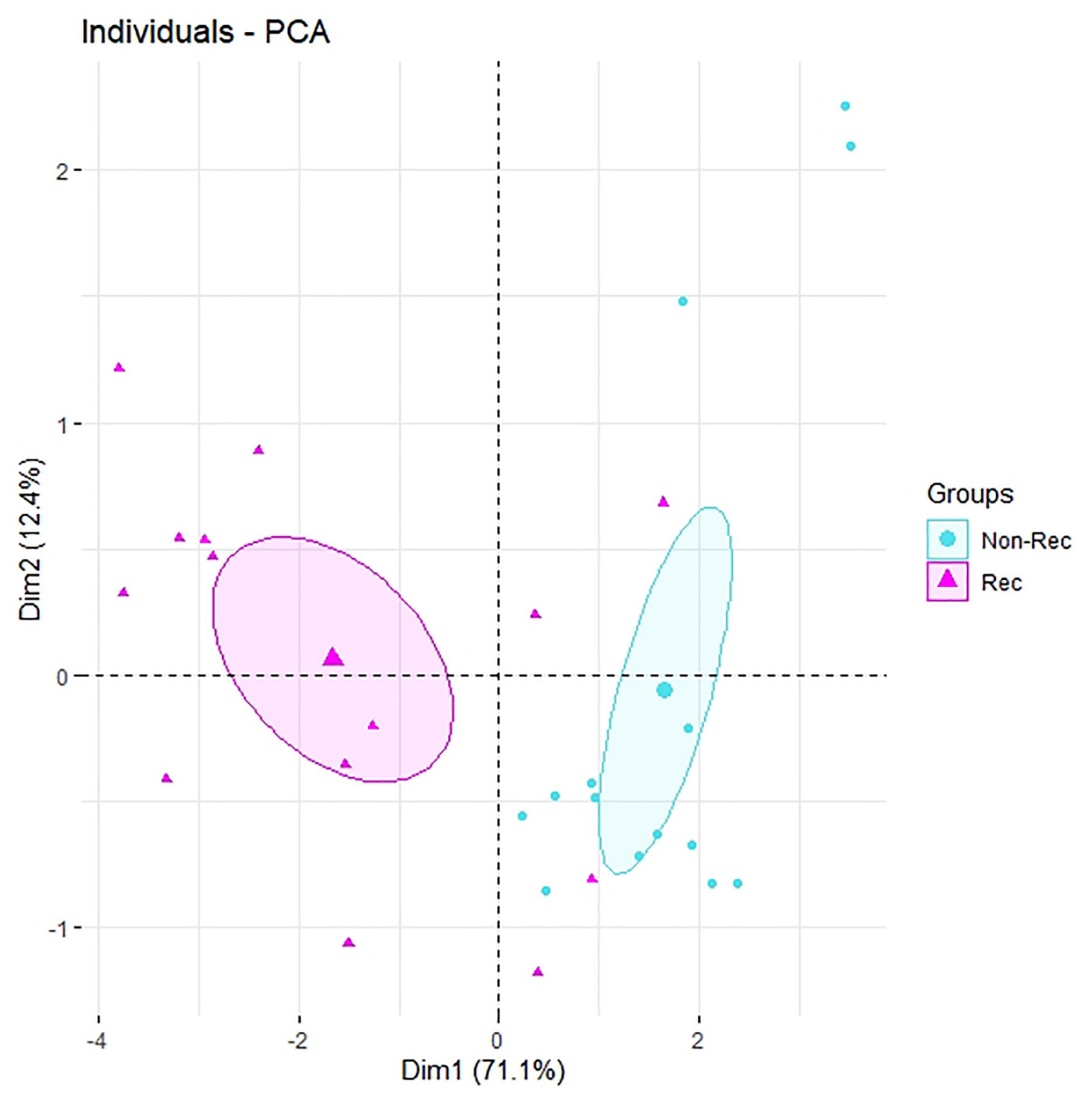
Two-dimensional scatter plot of the recurrent and non-recurrent regions based on the first two principal components derived from the seven significant features.

To reduce dimensionality, the principal component analysis was applied on the 7 significant features. Among the 7 computed principal components (PC), PC1 explained 71% of the variance and PC2 explained 12% of the variance. The 14 recurrent regions and 14 non-recurrent regions were then plotted as dots in the 2-dimensional PCA plot, where PC1 and PC2 yield x and y axes, respectively, as shown in Fig 5. In Fig 5, the recurrent regions and the non-recurrent regions do tend to form 2 clusters. Most of the non-recurrent regions were clustered closely with each other, while the recurrent regions were more spread out. This observation suggested that the recurrent regions contained more variations and were more heterogeneous than the non-recurrent regions. Except for patients 4, 11, 13 and 14, the rest of the recurrent regions could be well separated from the non-recurrent regions along PC1. Fig 2 showed the recurrent regions for patients 4, 11, 13 and 14 were large and taking up a big volume in the original tumor. This suggested that the MRI scans for patients 4, 11, 13 and 14 were performed after significant progression of the local recurrence and might be the reason why we could not separate them in the PCA plot.

## Discussion

To our knowledge, this is the first study of its kind to assess intra-tumoral radio-resistance using MRI radiomics features in patients with in-field recurrence after IMRT. The novelty of our study is that we performed intra-tumoral partitioning on the MRI at the time of diagnosis using information from MRI at the time of recurrence to characterize spatial heterogeneity. In this proof of concept study, we found a difference in the radiomics pattern between recurrent and non-recurrent regions within the NPC tumor. Notably, the results from the PCA showed that the recurrent regions contained more textural variations and were more heterogeneous than the non-recurrent regions. These results are hypothesis-generating and have potential clinical implications as we are currently unable to reliably predict the region of in-field recurrence from pre-treatment MRI. At present, a homogenous dose of radiotherapy is generally prescribed with the assumption that the whole tumor is homogenous. Recent technological advances in the field of radiation oncology have made it possible to escalate the dose safely. High risk tumor sub-regions associated with radio-resistance can then be targeted with a radiation boost or “dose painting” to potentially improve local control and patient survival.

There has been tremendous interest in radiomics research in NPC in the last few years. Previously, the majority of the radiomics studies in NPC have been focused on the analysis of the entire primary tumor as a whole to prognosticate disease outcomes or predict response to treatment. While the results from this approach supported the potential of radiomics in improving patient stratification for therapy, it assumes that the tumor is heterogenous but well mixed and ignores the regional variations within a tumor. [32,35,36] Studies from Ouyang et al and Zhang et al described their radiomics model based on pre-treatment contrast enhanced T1- and T2-weighted MRI which could predict disease progression in patients with NPC. [42] Zhang et al found textural features correlate not just to local failure but also to distant treatment failure. [37] Another study by Li et al showed that 8 radiomics features could differentiate in field recurrence from pre-treatment spectral attenuated inversion-recovery T2-weighted (SPAIR T2W) MRI. [34] Zhang et al produced a radiomics signature built with 11 features that outperformed conventional clinical variables in predicting local recurrence-free survival in patients with non-metastatic T4 NPC. [33] In a study of 120 NPC patients, Wang et al showed that the radiomics model could predict early response to induction chemotherapy.

However, the enthusiasm for the implementation of radiomics in routine practice should be tempered by a realization of its unique challenges. One of the main challenges includes the lack of standardization of image acquisition protocols. [43–45] Several studies have investigated the dependence of radiomics features on MRI field strength, imaging protocols, administration of contrast and scanner manufacturers in both living subjects and physical phantoms. These variations in acquisition parameters can introduce changes that are not due to underlying biologic effects on the images. Our study was not spared from such limitation. We acknowledge that the scans at diagnosis and at recurrence were performed in a different scanner at different time points with different scanning protocols. This was in part mitigated by including patients in a cohort with the same scanning protocol (between 2010–2012) to minimize the inconsistency within the pre-treatment MRI dataset. As such, radiomics data obtained from MRI acquired at a single institution using different image acquisition protocols, or acquired at different institutions with different scanners, may be affected by the heterogeneity of image acquisition, rather than reflecting the true difference in biological properties of the tumor, which constitute the biggest challenge for multicentric studies. Thus, the generalizability and reproducibility of radiomics modeling can be easily challenged and hamper its potential transfer to clinical practice. [46,47] In recent years, there have been many efforts in this direction by the Quantitative Imaging Network (QIN), sponsored by the National Cancer Institute (NCI), and the Quantitative Imaging Biomarker Alliance (QIBA), sponsored by the Radiological Society of North America (RSNA) to standardize imaging protocol for data sharing and advance the field of quantitative imaging.

Segmentation is a crucial step in the radiomics workflow as the radiomics features are extracted from the segmented volumes. In our study, the MRI images at the time of recurrence (without immobilization mask) were not perfectly matched to the MRI images at the time of diagnosis (with immobilization mask) given the difference in patient positioning. To minimize the geometrical uncertainties related to image fusion, we focused the region of image registration to a small volume of interest (VOI) centering at the recurrence area in the nasopharynx by using the pterygoid process and skull base as bony landmarks for matching in rigid registration and further fine-tuning. Accurate and robust tumor delineation is essential to ensure the reliability and reproducibility of the radiomics data. This is challenging since many NPC tumors have indistinct boundaries and are primarily treated with radiotherapy, thus neither the histological ground truth nor the robustness of the segmentation can be confirmed. Nonetheless, most authors would consider manual segmentation by experts the ground truth despite the lack of a standard for delineation and [48] being prone to inter-observer variability. To limit this problem in our study, all GTVr were contoured by a single experienced radiation oncologist. However, manual segmentation is a time-consuming task and not always feasible as radiomics analysis often requires very large datasets. There is an ongoing debate as to how much to rely on manual (solely by a human), automatic (solely by artificial intelligence, AI) or semi-automatic (human correction based on AI segmentation) segmentation. [49,50] Using machine learning techniques for auto-segmentation of malignant structures is an active area of research.

Our study has several other limitations. Most notably, the sample size was small, and hence the results of this exploratory study are hypothesis-generating, not confirming. Nonetheless, we hope that these preliminary results could encourage multi-institutional collaborative efforts for further verification with larger datasets. Secondly, the retrospective nature of data collection and retrieval of MRI at the time of local recurrence is an intrinsic limitation. Furthermore, the follow up MRI scans of four patients were performed after significant progression of the local recurrence as the recurrent regions occupied a big volume within the original tumor. Nonetheless, the treatment and follow up care schedule of NPC is protocolized given the high prevalence of NPC in our tertiary center and patients were followed up in a standardized manner. Although a prospective cohort study design is preferred, the protracted follow up periods required for recurrence to occur can require large resources. Third, we have only considered the MRI images from a single sequence (CE FS axial T1) in this study. Our results would likely improve when additional information from other sequences is integrated.

## Conclusions

In conclusion, our study showed that the radiomics features extracted from pre-treatment MRI could potentially reflect the difference between recurrent and non-recurrent regions within a tumor. The results from this study showed encouraging potential of MRI based radiomics for pre-treatment identification of intra-tumoral radio-resistance for selective dose escalation.

## Supporting information

S1 Appendix In-field recurrence. Axial slice pre-treatment MRI showing GTVr (gross tumor volume at recurrence) largely within the 95% isodose line.
(JPG)

S2 Appendix Image fusion. Left showing side by side overlay of MRI pre-treatment and MRI at recurrence showing GTV (light green): gross tumor volume at diagnosis (contoured on MRI pre-treatment); GTVr (red): gross tumor volume at recurrence (contoured on MRI at recurrence). Right showing all three GTV on MRI pre-treatment, GTVnr (yellow): non recurrent regions within GTV, obtained by subtracting the overlapping GTVr from the GTV (GTV-GTVr).
(JPG)
