# Supplementary figures and images for: Exploring MRI based radiomics analysis of intratumoral spatial heterogeneity in locally advanced nasopharyngeal carcinoma treated with intensity modulated radiotherapy
Source: PLoS One. 2020 Oct 5;15(10):e0240043. doi: 10.1371/journal.pone.0240043 (PMC7535039; doi:10.1371/journal.pone.0240043)

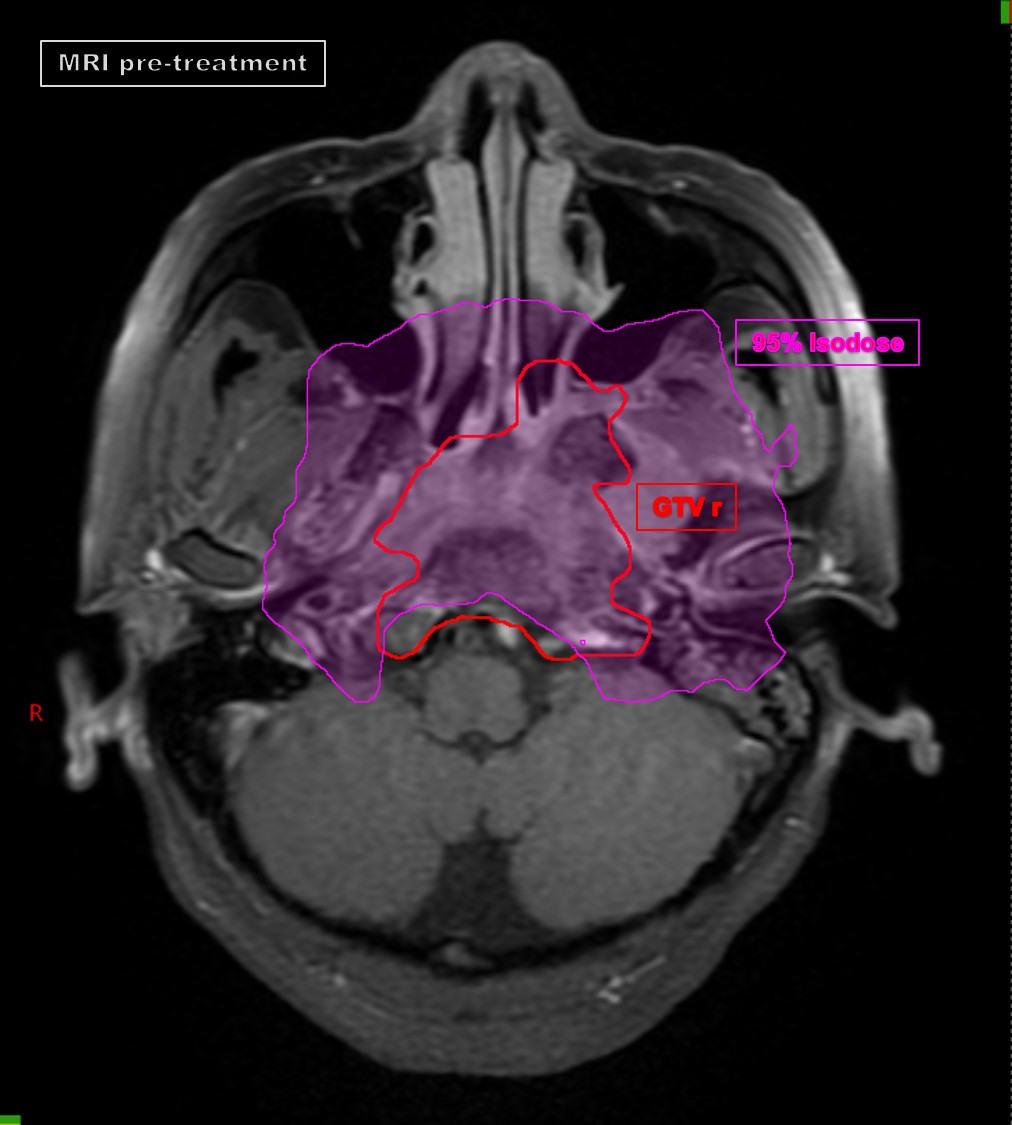

Supplement: S1 Appendix — Axial slice pre-treatment MRI showing GTVr (gross tumor volume at recurrence) largely within the 95% isodose line. (JPG) [file pone.0240043.s001.jpg]

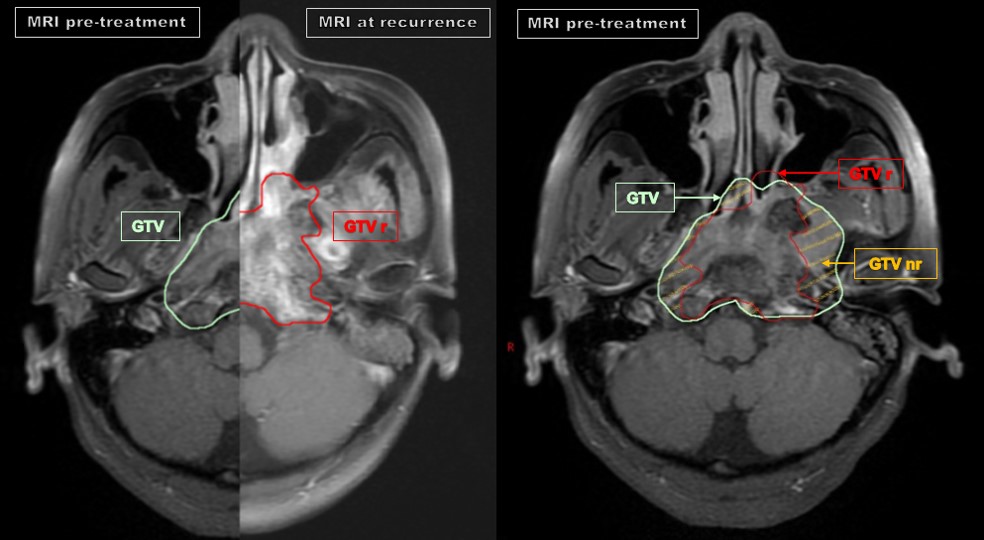

Supplement: S2 Appendix — Left showing side by side overlay of MRI pre-treatment and MRI at recurrence showing GTV (light green): gross tumor volume at diagnosis (contoured on MRI pre-treatment); GTVr (red): gross tumor volume at recurrence (contoured on MRI at recurrence). Right showing all three GTV on MRI pre-treatment, GTVnr (yellow): non recurrent regions within GTV, obtained by subtracting the overlapping GTVr from the GTV (GTV-GTVr). (JPG) [file pone.0240043.s002.jpg]
